# Transcriptomic Analysis of the Diamondback Moth Under Exposure to the Juvenile Hormone Esterase (JHE) Inhibitor 3-Octylthio-1,1,1-trifluoro-2-propanone (OTFP)

**DOI:** 10.3390/insects16111152

**Published:** 2025-11-11

**Authors:** Yingbo Wang, Xiying Wang, Yubin Lin, Shun Zheng, Jianrong Qiu, Jinheng Gao, Xiaojun Gu, Jingfei Huang

**Affiliations:** 1 College of Plant Protection, Fujian Agriculture and Forestry University, Fuzhou 350002, China; 2 State Key Laboratory of Agriculture and Forestry Biosecurity, Fuzhou 350002, China

**Keywords:** *Plutella xylostella*, RNA-seq, OTFP, juvenile hormone esterase, insect hormone pathway

## Abstract

Targeting juvenile hormone esterase (JHE) is an emerging strategy to combat the broadly resistant pest, *Plutella xylostella*; this study employed transcriptomics to investigate the sublethal effects of the JHE inhibitor OTFP, revealing a non-monotonic dose response characterized by stronger transcriptional changes at lower concentrations, resulting in low mortality, prolonged pupation time, and increased pupal weight. The results from the Differentially Expressed Genes (DEGs) analysis revealed that the core effect of OTFP is the persistent perturbation of the “insect hormone biosynthesis” pathway and altered expression of components of the JH/20E axis; to cope with this stress, the larvae exhibited a dual defense associated with compensatory upregulation of JH-degrading enzyme genes to attempt to restore hormone homeostasis, and the activation of a broad-spectrum detoxification network to clear the compound. More critically, the developmental delay resulting from endocrine disruption KEGG-enriched growth-related pathways (amino-acid and central-carbon metabolism; ribosome biogenesis; aminoacyl-tRNA biosynthesis), consistent with a growth-permissive milieu during prolonged feeding. This study therefore elucidates a novel integrative regulatory network that links endocrine disruption, detoxification, and compensatory growth, revealing a complex physiological trade-off strategy in this pest that sacrifices developmental tempo for survival.

## Simple Summary

The diamondback moth is a major global pest with widespread pesticide resistance. Our previous research found that OTFP, a juvenile hormone esterase inhibitor, could extend the larval stage, delay the pupation, and increase the pupal weight of the diamondback moth. To elucidate the underlying mechanism, this study employed transcriptomic sequencing to comprehensively analyze expression changes in all genes responding to the OTFP exposure. Our findings reveal that OTFP disrupts the crucial JH/20E hormone pathways that govern development. In response, the moth primarily attempts to restore balance through the compensatory expression of genes within the hormone pathway, in addition to activating detoxification genes. Unexpectedly, the prolonged larval period was accompanied by transcriptional remodeling of KEGG-enriched growth-related programs—especially amino acid and central-carbon metabolism together with ribosome biogenesis and aminoacyl-tRNA biosynthesis—which may allow larvae to convert developmental delay into biomass accumulation. This research reveals a complex trade-off strategy within the insect, offering new molecular insights into the intricate adaptive processes by which insects cope with short-term, xenobiotic-induced disruptions to their endogenous hormone balance.

## 1. Introduction

Insect hormones control a wide range of physiological behaviors and life processes, including growth, development, reproduction, and migration [1]. In holometabolous insects, such as lepidoptera, diptera and coleptera, molting and metamorphosis are precisely regulated by two key hormones: the steroid 20-hydroxyecdysone (20E) and the sesquiterpenoid juvenile hormone (JH) [2,3]. The prothoracic gland synthesizes and secretes ecdysone (E) into the hemolymph, where peripheral tissues convert it to the active hormone 20-hydroxyecdysone (20E) via Shade/CYP314A1, which primarily governs larval molting, ovarian development, metamorphosis and postembryonic growth [4,5,6,7]. The signaling pathway of 20E was first characterized in *Drosophila melanogaster*, where the ecdysone receptor (EcR) forms a heterodimer with the ultraspiracle protein (USP). This EcR/USP complex binds to ecdysone response elements (EcREs) in the genome, directly regulating the expression of downstream target genes such as *E74*, *E75*, *Broad-complex (BrC)*, and *E93* [8,9,10]. In contrast, JH is synthesized and secreted by the corpora allata, functioning to maintain larval characteristics, promote larval growth, and modulate reproductive behavior [11,12]. The molecular mechanism of JH signaling pathway involves the Methoprene-tolerant (Met) protein, which binds JH with high affinity. Subsequent studies identified a paralogous gene, *Gce*, in *Drosophila*, revealing partial functional overlap with Met. Upon JH binding, Met (or Gce) interacts with the bHLH-PAS protein Taiman (Tai) to form a transcriptional complex. This complex activates *Krüppel homolog 1 (Kr-h1)* by binding with JH response elements (JHREs) in its core promoter segment. *Kr-h1* is a key downstream transcription effector of JH that suppresses metamorphosis by modulating 20E-mediated signaling, thereby preserving larval status [13,14,15].

While the Met-Taiman-Kr-h1 pathway conducts the JH signaling cascade transduction and significantly dictates the developmental process, the biological potency of JH is ultimately controlled by its spatiotemporal titers [2,16], which are coordinately regulated through three core processes: (1). Biosynthetic Regulation, JH biosynthesis in the corpora allata integrates multiple upstream inputs—including insulin/TOR signaling and neuropeptides such as allatostatins and allatotropins; the relative contributions of these inputs vary by species and physiological context. Studies suggest insulin signaling influences JH production, though its dominance is debated, with focus on JH’s developmental effects and a broader overview of JH regulation [17,18]; (2). Transport and Homeostasis, JH transport via hemolymph JH-binding proteins (JHBPs) critically influences hormone bioavailability, primarily by protecting JH from esterase hydrolysis and mediating its transport in hemolymph [19]. Enzymatic Degradation, two hydrolytic enzymes dominate JH catabolism: juvenile hormone esterase (JHE), which cleaves the methyl ester from JH to form juvenile hormone acid (JHa), and juvenile hormone epoxide hydrolase (JHEH), which hydrolyzes the epoxide moiety of JH to form juvenile hormone diol (JHd) [2,20]. JHE exhibits functional versatility due to its dual localization (extracellular hemolymph and intracellular compartments), capacity to degrade both free and JHBP-bound JH, and essential role in JH clearance during metamorphosis [21,22]. Dysregulation of JHE disrupts developmental timing, as demonstrated by precocious metamorphosis in JHE-overexpressing silkworms [23]. Enzymatic degradation via JHE is considered as a critical checkpoint for the precious regulation of normal development. JHE not only ensures timely metamorphosis but also represents a potential vulnerability for targeted pest control [24,25]. Based on the important roles of JHE on insect growth and development, previous reports have been focused on the potential of JHE inhibitor on pest control [26].

To date, among the identified JHE inhibitors, O-ethyl-S-phenyl phosphoramidothiolate (EPPAT) and trifluoromethyl ketones (TFMKs), are the two most selective and effective options. The distinguishing feature of these two types of compounds lies in their distinct modes of action. The inhibition of JHE by EPPAT is irreversible, whereas the effect of TFMKs on JHE is competitive and reversible [20,27]. A notable example of a competitive and reversible inhibitor is OTFP (3-octylthio-1,1,1-trifluoro-2-propanone), which demonstrates a slow and tight binding mechanism [20,28]. OTFP has been shown to disrupt the mating behavior of corn stem borers and induce infertility in the majority of their eggs. Additionally, OTFP has demonstrated a significant inhibitory effect on the growth of larvae from *Spodoptera littoralis* and *Sesamia nonagrioides* at low doses. This effect is associated with dose-related changes and is linked to starvation-related mortality in the insects at higher doses. Furthermore, adult insects treated with OTFP displayed a reduced response to sex pheromones. This diminished response may be due to the incomplete detoxification of OTFP by the detoxification enzymes present in the insect gut, leading to prolonged effects. However, OTFP did not influence the production of sex pheromones in female insects, indicating that its impact on adult behavior arises from long-term interference with the internal physiology of these insects [29].

The diamondback moth (*Plutella xylostella*) (Lepidoptera: Plutellidae), a globally invasive pest originating from South America, currently infests cruciferous crops in 140 countries [30]. It is characterized by rapid life cycles (≤18 days), multivoltine reproduction (≥20 generations annually), and exceptional adaptability to xenobiotics (such as pesticides) and extreme environments [31,32]. *P. xylostella* has evolved unprecedented resistance to all major insecticide classes, incurring annual economic losses estimated at USD 4–5 billion [33,34,35,36]. Given the rapid development of resistance in *P. xylostella*, there is an urgent need for alternative pest control strategies. JHE inhibitors, such as TFMKs, offer a promising avenue for targeting the JH signaling pathway, which is essential for the development and reproduction of insects. Based on our previous studies [24,26], OTFP reduced hemolymph JHE activity relative to time-matched controls at both time points (24 h: 4.66 vs. 6.66/6.15 mM·min^−1^·mL^−1^; 48 h: 6.89 vs. 9.73/9.67 mM·min^−1^·mL^−1^), and down-regulated JHE gene (Px004817) expression (ΔCt decreased by 3.2-fold) in *P. xylostella*, leading to delayed larval development (prolonged pupation time by 8.37 h) and increased pupal weight (7.35 mg vs. 6.27 mg in controls). Further evidence indicated that OTFP disrupts the juvenile hormone (JH) signaling pathway, resulting in abnormal larval-pupal transition, though the underlying molecular mechanisms remain unclear. To systematically elucidate how OTFP modulates JH signaling and developmental processes, the present study performs transcriptome sequencing to analyze genome-wide gene expression changes in *P. xylostella* during critical developmental stages (the fourth instar to prepupal phase) under the exposure of low-dose OTFP, and focuses on the dynamic regulation network of the JH signaling pathway and its cross-talk with ecdysteroid signaling. The findings of this study can provide a theoretical foundation for management strategies of *P. xylostella* based on hormonal regulation.

## 2. Materials and Methods

### 2.1. Insect Rearing

The strain of diamondback moth, *P. xylostella*, used in this study was sourced from a laboratory colony maintained in Fujian Agriculture and Forestry University Fuzhou, China (established prior to 2021) and reared for multiple generations under pesticide-free conditions. Larvae were reared on white radish seedlings (*Raphanus sativus* L.), and adults were supplied with a 10% (*w*/*v*) honey solution as a nutritional supplement. All rearing and experimental procedures were conducted in an artificial climate chamber maintained at a temperature of 25 ± 1 °C, a relative humidity of 75% ± 5%, and a photoperiod of 16:8 h (Light:Dark).

### 2.2. Chemical Treatment and Bioassay

The juvenile hormone esterase inhibitor, 3-octylthio-1,1,1-trifluoro-2-propanone (OTFP) (>98% purity), was purchased from Doly Medicinal Chemistry Co., Ltd. (Shanghai, China). Two sublethal concentrations were selected for this study based on preliminary toxicity screening: 1325 mg/L (designated as TA) and 2650 mg/L (designated as TB). In our previous screening assay, fourth-instar larvae treated for 48 h with OTFP at concentrations of 662.5, 1325, and 2650 mg/L exhibited mortality rates of 4.2%, 2.9%, and 4.2%, respectively. As all mortality rates were below 5%, these concentrations were confirmed to be sublethal. The treatment solutions were prepared in sterile distilled water containing 0.5% ethyl acetate and 0.05% Triton-100; this solvent was also used as the control (CK). Bioassays were conducted using a standard leaf-dip method. Healthy, actively feeding early fourth-instar larvae, approximately 6 mm in length, were selected and starved for 6 h prior to the assay. Leaf discs (3 cm in diameter) were punched from fresh radish leaves, dipped into the respective treatment or control solutions for 10 s, air-dried on filter paper, and placed in Petri dishes (10 cm in diameter) lined with moistened filter paper. Ten starved larvae were introduced into each Petri dish. Each treatment was performed with three independent biological replicates.

### 2.3. Sample Collection and RNA Extraction

RNA was extracted from date-one fourth-instar larvae of the control (CK) and experimental groups (TA and TB) at 24 and 48 h post-OTFP treatment, corresponding to early and middle stages of the final larval period. For each time point and treatment group, three independent biological replicate samples were collected, with each replicate consisting of 15 pooled larvae. Collected samples were immediately flash-frozen in liquid nitrogen and subsequently stored at −80 °C for downstream transcriptomic and qRT-PCR analyses. Total RNA was extracted from each sample using the TRIzol method. The concentration, purity (OD260/280 ratio between 1.8 and 2.1), and integrity of the extracted RNA were assessed using a NanoDrop spectrophotometer (KAIAO, Beijing, China) and agarose gel electrophoresis (Sigma-Aldrich, Shanghai, China).

### 2.4. cDNA Library Construction and Transcriptome Sequencing

High-quality total RNA samples were submitted to Biomarker Technologies (Beijing, China) for cDNA library construction and sequencing. Briefly, mRNA was enriched from total RNA using oligo(dT)-attached magnetic beads, followed by random fragmentation. First- and second-strand cDNA were synthesized using the fragmented mRNA as a template. The purified double-stranded cDNA underwent end-repair, A-tailing, sequencing adapter ligation, and PCR enrichment to complete the library construction. After passing quality control, the libraries were sequenced on an Illumina HiSeq 4000 platform (Illumina, BioMarker, Beijing, China). The raw sequencing data have been deposited in the NCBI under BioProject accession number PRJNA1320849, and the data quality assessment is summarized in Table S1 of Supplementary File.

### 2.5. Bioinformatic Analysis

**Figure 1:**
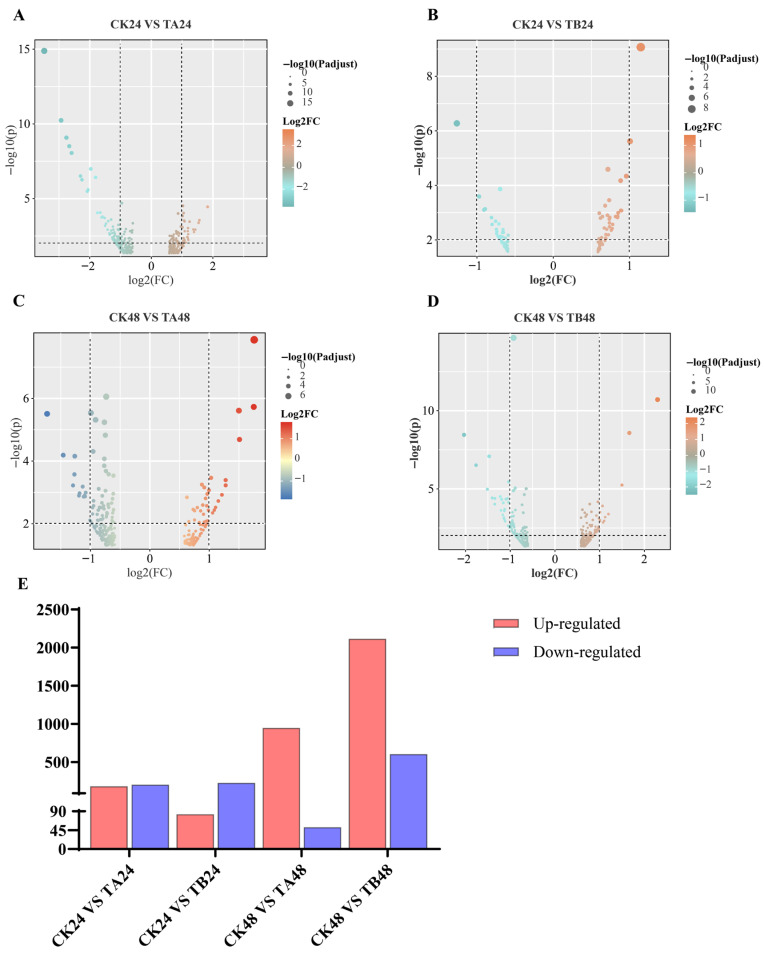
Overview of differentially expressed genes (DEGs) in *P. xylostella* larvae following OTFP treatment. (**A**–**D**) Volcano plots showing the distribution of DEGs in the four main comparison groups. Each point represents a single gene. The x-axis indicates the log2(Fold Change), and the y-axis represents the statistical significance (−log10 *p*-value). The color gradient corresponds to the magnitude of the fold change (red: upregulation; blue/green: downregulation), while the size of each point is proportional to *p*-value. The specific comparisons are: (**A**) CK24 vs. TA24, (**B**) CK24 vs. TB24, (**C**) CK48 vs. TA48, and (**D**) CK48 vs. TB48. (**E**) Bar chart summarizing the total number of significantly upregulated (red bars) and downregulated (blue bars) DEGs for each comparison. DEGs were defined by the criteria of an absolute log2(Fold Change) ≥ 1 and an *p* < 0.05.

**Figure 2:**
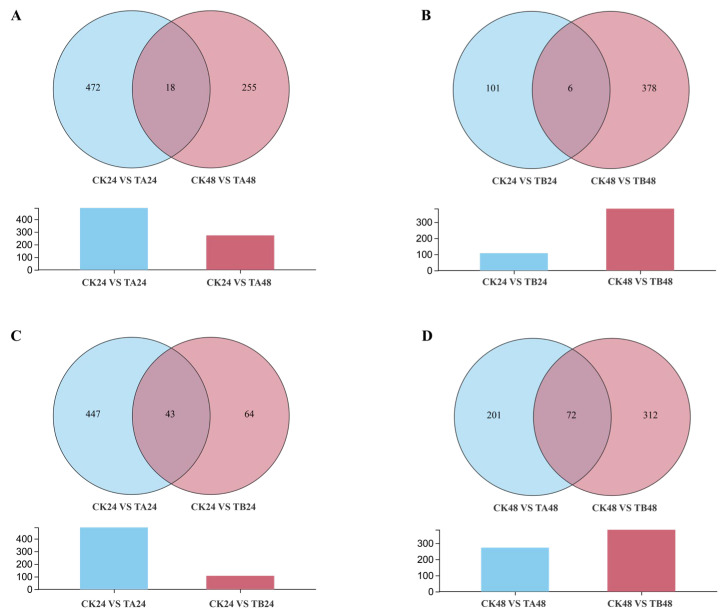
Venn diagrams summarizing the unique and shared significant transcripts (*p* < 0.05) among comparisons. (**A**) CK24 vs. TA24 vs. CK48 vs. TA48; (**B**) CK24 vs. TA24 vs. CK24 vs. TB24; (**C**) CK24 vs. TB24 vs. CK48 vs. TB48; (**D**) CK48 vs. TA48 vs. CK48 vs. TB48. Note: sets include all genes with *p* < 0.05 without a fold-change cutoff, hence termed significant transcripts.

Raw sequencing reads were processed to remove adapter sequences and low-quality reads, yielding high-quality clean reads. The clean reads were then aligned to the *P. xylostella* reference genome (*Plutella xylostella*. lepbase_v4) using HISAT2 (v2.1.0) software. Subsequently, transcript assembly was performed on the aligned reads using StringTie (v2.2.3) software. Differentially expressed genes (DEGs) were identified using the DESeq2 package in R, with screening criteria set at |Log2(FoldChange)| ≥ 1 and *p*-value < 0.05. For volcano plots and DEG counts (Figure 1), we used |log2FC| ≥ 1 & *p* < 0.05. For Venn diagrams (Figure 2), we summarized all transcripts meeting statistical significance (*p* < 0.05) irrespective of fold-change; therefore, these sets are referred to as ‘significant transcripts (*p* < 0.05)’ rather than DEGs. The identified DEGs were subjected to Gene Ontology (GO) and Kyoto Encyclopedia of Genes and Genomes (KEGG) pathway enrichment analyses using a hypergeometric test, with a *p*-value < 0.05 considered as the threshold for significant enrichment.

### 2.6. Data Visualization and Statistical Analysis

Data visualization was primarily performed using R (v4.1.2) and GraphPad Prism 9. Volcano plots and Venn diagrams were generated from the DESeq2 output using relevant R packages. Bubble charts for GO and KEGG enrichment analyses were created using the ggplot2 package in R. Heatmaps of gene expression were generated using the pheatmap package in R, based on Z-score normalized log2(FPKM + 1) values. Bar charts for qRT-PCR data were created and statistically analyzed using GraphPad Prism 9.

### 2.7. Quantitative Real-Time PCR (qRT-PCR) Validation

To validate the reliability of the transcriptome data, a total of 13 DEGs involved in juvenile hormone signaling and detoxification pathways were selected for qRT-PCR analysis (the primers used for validation are listed in Table S2 of Supplementary File). After treatment with OTFP at different time intervals, total RNA of samples was extracted as described previously. First-strand cDNA was synthesized using the NovoScript^®^ Plus All-in-one 1st Strand cDNA Synthesis SuperMix (gDNA Purge) kit (Novoprotein, Suzhou, China). The qPCR reactions were performed using the TransStart^®^ Tip Green qPCR SuperMix kit (TransGen, Beijing, China) on a real-time PCR instrument. Three biological replicates were analyzed for each gene. The house-keeping gene *Ef (Elongation factor)* was used as control for normalization, and the relative expression levels were calculated using the 2^−ΔΔCt^ method to assess the accuracy of the sequencing data. Significant differences between two groups were determined by Student’s *t*-test (* *p* ≤ 0.05; ** *p* ≤ 0.001), while comparisons among multiple groups were performed using one-way analysis of variance (ANOVA) followed by Tukey’s post hoc test. All data are presented as mean ± standard deviation (SD).

## 3. Results

### 3.1. Sequencing Data Summary and Quality Assessment

To investigate the transcriptomic response of *P. xylostella* to the JHE inhibitor OTFP, a total of 21 cDNA libraries from control and treatment groups of fourth-instar larvae were constructed and sequenced. The sequencing generated a total of 200.70 Gb of clean data. On average, each sample yielded over 6.52 Gb of clean data, with the percentage of Q30 bases consistently exceeding 94.08% and the GC content averaging approximately 51%. The clean reads from each library were mapped to the designated *P. xylostella* reference genome, with alignment efficiencies ranging from 69.59% to 76.95% (detailed in Table S1). Collectively, these metrics demonstrate that the transcriptome dataset is of high quality and provides sufficient depth and coverage for subsequent differential expression and functional analyses.

### 3.2. OTFP Treatment Induced Extensive Transcriptional Reprogramming in Fourth-Instar P. xylostella

The transcriptional response of *P. xylostella* to OTFP was both dynamic and complex, exhibiting a non-linear dependence on dose and time. In the initial phase (24 h), the response was predominantly suppressive. As revealed by a combined analysis of the volcano plots and bar chart, this was evident in both the low-concentration (CK24 vs. TA24) and high-concentration (CK24 vs. TB24) treatment groups, where down-regulated genes significantly outnumbered up-regulated ones (Figure 1A,B,E). However, after 48 h of exposure, this pattern underwent a fundamental reversal, shifting to a large-scale gene activation profile. This shift was particularly pronounced in the high-concentration group (CK48 vs. TB48), which showed an overwhelming activation event with 2719 total DEGs identified; of these, the number of up-regulated genes was more than threefold higher than that of down-regulated ones (Figure 1D,E).

To dissect the dose- and time-dependent dynamics of the transcriptional response, a Venn diagram analysis of the significant transcripts was performed. This analysis revealed a complex, non-linear response pattern. First, the temporal dynamics of the response exhibited a marked difference between the two concentrations. Under low-concentration treatment, the number of unique DEGs decreased from 472 at 24 h to 255 at 48 h (Figure 2A). In contrast, under high-concentration treatment, the number of unique significant transcripts increased sharply from 101 at 24 h to 378 at 48 h (Figure 2C). Second, the dose effect also reversed over time. At 24 h post-treatment, a substantially greater number of unique significant transcripts were induced by the low-concentration treatment (TA24; 447 genes) compared to the high-concentration treatment (TB24; 64 genes) (Figure 2B). Notably, this dose-effect was reversed at 48 h post-treatment, at which point the high-concentration group (TB48; 312 genes) induced more unique significant transcripts than the low-concentration group (TA48; 201 genes) (Figure 2D). Furthermore, the overlap of responsive genes between the 24 h and 48 h time points was minimal for both concentrations (18 and 6 genes, respectively), indicating that the transcriptional programs activated at each stage are highly specific.

### 3.3. Functional Enrichment Analysis Revealed Dynamic Toxicological Effects Centered on Endocrine Disruption

To elucidate the biological implications of the extensive transcriptional changes, Gene Ontology (GO) and KEGG pathway enrichment analyses were performed on the differentially expressed genes (DEGs) from each comparison group. The results indicate that the response of *P. xylostella* to OTFP is a complex process that evolves dynamically with dose and time. This response is centered on the disruption of the endocrine system and is associated with cellular stress and detoxification defenses, as supported by gene-level changes.

**Figure 3:**
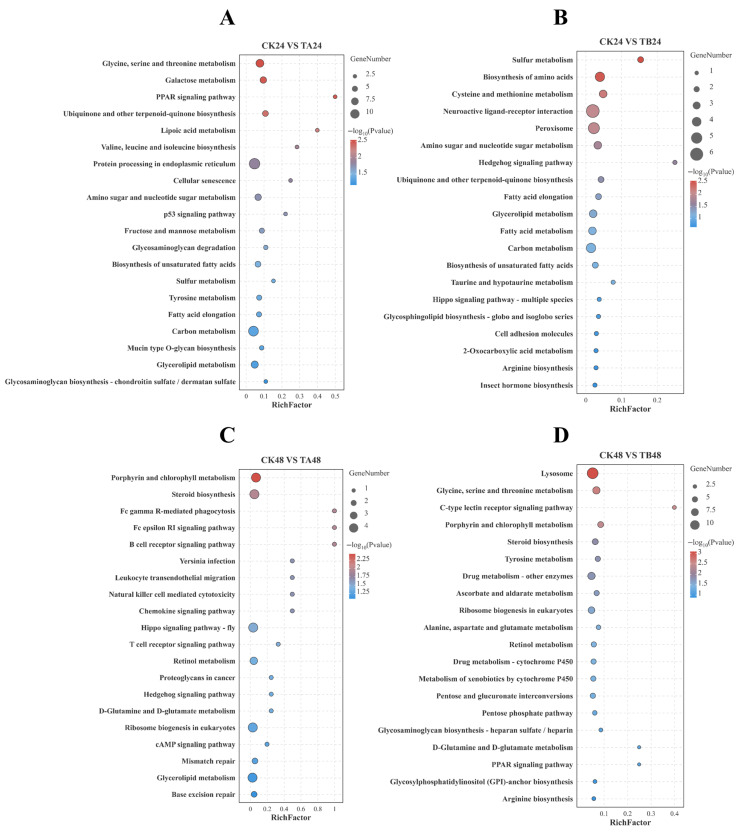
KEGG pathway enrichment analysis of differentially expressed genes (DEGs) in different treatment groups. KEGG pathway enrichment analysis of differentially expressed genes (DEGs) in different treatment groups. The bubble plots show the main signaling pathways enriched in the four main comparison groups (**A**–**D**). The size of the bubble represents the number of enriched genes, and the color represents the significance of the *p*-value.

**Figure 4:**
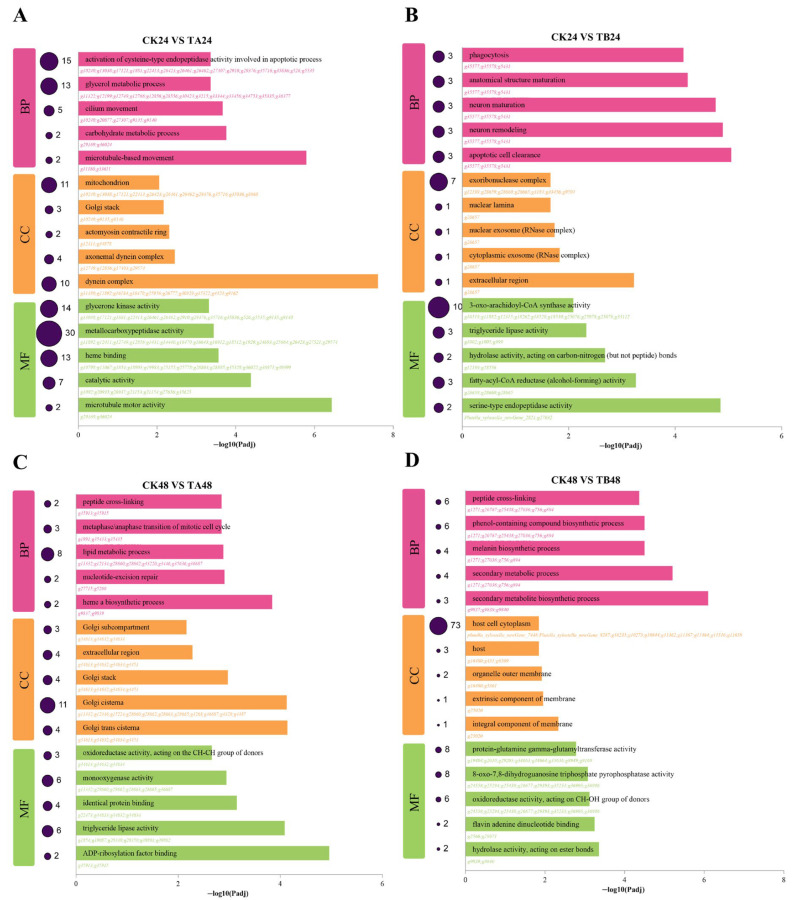
GO functional enrichment analysis of differentially expressed genes (DEGs) in different treatment groups. GO functional enrichment analysis of differentially expressed genes (DEGs) in different treatment groups. The bar charts show the top GO terms in Biological Process (BP), Cellular Component (CC), and Molecular Function (MF) for the four main comparison groups (**A**–**D**). The circles in the figure represent the number of genes enriched in the relevant processes.

**Figure 5:**
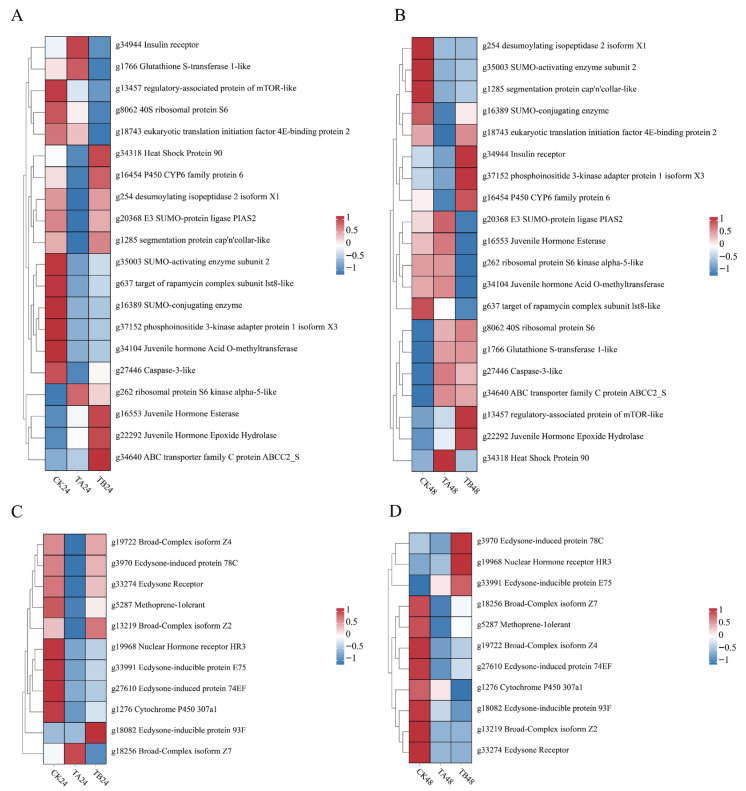
Expression profiles of genes involved in juvenile hormone (JH) metabolism, mTOR signaling, and the JH/20E signaling pathway following OTFP treatment. The heatmap shows the expression levels of differentially expressed genes (red indicates upregulation; blue indicates downregulation) following OTFP treatment. Panels (**A**) and (**B**) show the expression profiles of genes related to JH metabolism and the mTOR pathway at 24 and 48 h, respectively, while (**C**) and (**D**) show the profiles of genes in the JH/20E signaling pathway at the corresponding time points. CK, TA, and TB represent the control, low-concentration, and high-concentration treatment groups, respectively.

In the early stage (24 h), the transcriptional response pattern showed a non-monotonic dose-associated shift, with a stronger response in the low-concentration group (TA24) than in the high-concentration group (TB24), evidenced by more significantly enriched KEGG pathways and GO terms (*p* < 0.05), indicative of a non-monotonic dose response. Under low-concentration treatment (TA24), the organism displayed widespread metabolic disruption, with KEGG analysis showing significant enrichment of DEGs in the “PPAR signaling pathway” and various amino acid and lipid metabolism pathways (Figure 3A). This was accompanied by a distinct biological process enrichment in GO analysis, which pointed to the “activation of cysteine-type endopeptidase activity involved in apoptotic process” (Figure 4A). In contrast, the high-concentration treatment (TB24) induced a more direct disruption of the endocrine system. In contrast, the high-concentration treatment (TB24) induced a more direct disruption of the endocrine system. The “Insect hormone biosynthesis” pathway was not significantly enriched (*p* = 0.07, Figure 3B) but suggests a biologically relevant trend. The lack of statistical significance is likely a technical artifact of incomplete KEGG pathway annotation for *P. xylostella*, which fails to map many of the known, critical hormone-related genes. We support evidence for endocrine disruption at 24 h primarily through direct gene-level analysis (Figure 5, e.g., *JHE, JHEH, JHAMT, Met, EcR*), which bypasses this database limitation. This severe endocrine disruption was associated with significant cellular damage, as GO enrichment results showed the activation of biological processes such as “apoptotic cell clearance” and “neuron remodeling” (Figure 4B).

In the later stage (48 h), the response pattern showed a non-monotonic dose-associated divergence, with the low-concentration group (TA48) exhibiting more significantly enriched KEGG pathways and GO terms (*p* < 0.05) than the high-concentration group (TB48). At the low concentration (TA48), the response shifted towards cellular repair and signal recalibration. KEGG analysis revealed enrichment of pathways related to ecdysteroids (“Steroid biosynthesis”) (Figure 3C), while GO analysis indicated enrichment of “nucleotide-excision repair” and “peptide cross-linking” (Figure 4C). This suggests an attempt by the organism to repair damage and stabilize its endocrine and developmental signaling networks. Under high-concentration, prolonged treatment (TB48), however, the transcriptional response was predominantly characterized by the activation of broad-spectrum detoxification pathways. The KEGG analysis showed that DEGs were highly and significantly enriched in the core xenobiotic metabolism pathways, including “Drug metabolism and cytochrome P450” (Figure 3D). This clearly indicates that activating a broad-spectrum detoxification network centered on P450s and GSTs is the primary defense strategy under high-dose stress in the later stage. This conclusion was supported by the GO enrichment results, which showed significant enrichment in the Molecular Function category for “hydrolase activity, acting on ester bonds,” a function highly relevant to both detoxification and the compensatory expression of esterases (Figure 4D).

In summary, the functional enrichment analysis indicates that the early effects of OTFP involve a dose-dependent endocrine disruption and associated apoptosis. In the later stage, the organism’s response is altered according to the intensity of the stress: shifting towards repair and signal recalibration at low doses, while launching a large-scale detoxification program to cope with sustained chemical stress at high doses.

### 3.4. OTFP Induces an Integrative Molecular Response from Hormonal Imbalance to Suppression of Developmental Signaling

OTFP treatment induced a multi-level molecular response in *P. xylostella*. At its core, OTFP fundamentally disrupted the insect’s endocrine system. In response to JH accumulation, the organism compensatorily upregulated the expression of *juvenile hormone esterase* (*JHE*) and *juvenile hormone epoxide hydrolase* (*JHEH*), while the transcription of the rate-limiting biosynthetic enzyme, JHAMT, was feedback-inhibited (Figure 5A,B). This endocrine dysregulation is associated with a complex stress defense and metabolic reprogramming response. The organism activated a detoxification network, including the upregulation of a Phase I cytochrome P450 (CYP) gene, alongside dynamic regulation of a Phase II glutathione S-transferase (GST) and transcriptional suppression of a Phase III ABC transporter. The lack of synergistic upregulation of the master regulator CncC and the stress protein Hsp90 highlights the complexity of this regulatory network (Figure 5A,B). As another face of the response, growth-signaling components (IIS–PI3K/AKT) and translation-associated effectors (e.g., RPS6) showed transcript increases (Figure 5B). Because pathway output is primarily phosphorylation-controlled, these mRNA changes are interpreted as transcriptional remodeling rather than definitive activation.

Ultimately, the JH imbalance induced by OTFP led to the systemic suppression of downstream signaling factors that regulate metamorphosis, and the transcript level of the core JH pathway receptor, *Met*, was upregulated. Concurrently, the 20E signaling pathway underwent a comprehensive transcriptional suppression, spanning multiple regulatory levels from the upstream synthesis enzyme (*CYP307A1*) and the core receptor (*EcR*) to various primary and late response genes (e.g., *BRC*, *E75*) (Figure 5C,D). In summary, OTFP disrupts JH metabolic homeostasis, leading to a cascade of effects that suppress key hormonal signaling pathways and ultimately block the critical processes governing metamorphosis.

### 3.5. qRT-PCR Validation of Transcriptome Data

**Figure 6:**
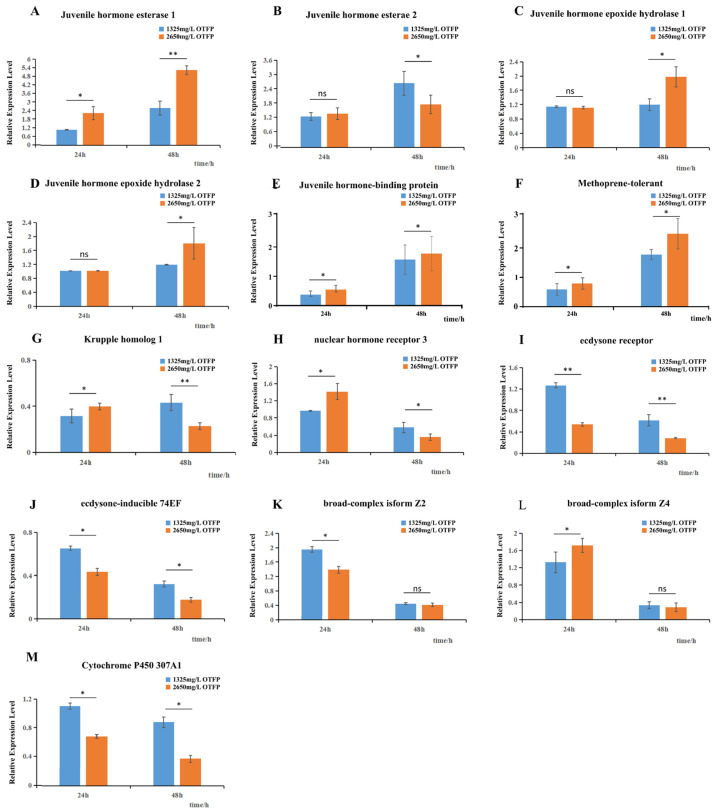
Dynamic expression patterns of 13 key genes under different treatments analyzed by qRT-PCR. Dynamic expression patterns of 13 key genes under different treatments, as determined by qRT-PCR ((**A**–**M**): *JHE1, JHE2, JHEH1, JHEH2, JHBP, MET, Kr-h1, HR3, ECR, 74EF, BrC-Z2, BrC-Z4, CYP307A1*). The bar charts show the relative expression levels of 13 selected genes under different concentrations and time points. The x-axis represents the sampling time after treatment, and the y-axis shows the relative expression levels of each gene normalized to the house-keeping gene *ef* at each time point. Error bars represent the standard deviation of three biological replicates. Asterisks indicate statistically significant differences compared with the control group (*p* < 0.05, *; *p* < 0.01, **), and ns indicates no significant difference (*p* ≥ 0.05).

To precisely quantify the dose- and time-dependent effects of OTFP, the expression dynamics of 13 key regulatory genes were analyzed by qRT-PCR, providing validation for the transcriptomic data. The results accurately confirmed the pattern of compensatory gene expression. For instance, the transcript levels of the JH degradation enzyme *JHE* were consistently upregulated at both 24 h and 48 h (Figure 6A,B), with a more pronounced response at the higher concentration (Figure 6A). The upregulation of another catabolic enzyme, *JHEH1*, was primarily observed at 48 h under high concentration (Figure 6C,D), indicating distinct temporal dynamics in the compensatory responses of different enzymes. Components of the JH and 20E pathways showed transcriptional changes (Figure 6F–M). Notably, Met and EcR decreased across treatments (Figure 6F–M). For instance, their core components—the JH receptor *Met* and the 20E receptor *EcR*—exhibited sustained and dose-dependent downregulation (Figure 6F,I). Consistent with this, the transcriptional suppression of key downstream 20E pathway effectors, such as *HR3* and *BRC-Z4*, was maximal at 48 h (Figure 6H,L), a time point that corresponds closely with the observed developmental delay.

## 4. Discussion

Within the conventional toxicological framework, sublethal chemical stressors, such as endocrine-disrupting insecticides and insect growth regulators (IGRs), consistently trigger a cascade of adverse physiological outcomes [37,38]. These effects initially manifest as feeding inhibition and behavioral impairment, advancing to disruptions in metabolic homeostasis and quasi-dormancy or developmental stasis [39,40,41], ultimately resulting in metamorphic abnormalities (e.g., pupal deformities) and reduced fitness [38,42]. Mechanistically, juvenile hormone (JH) analogs (e.g., pyriproxyfen) and juvenile hormone esterase (JHE) inhibitors perturb the hormonal homeostasis of the JH/20-hydroxyecdysone (20E) axis [20,27,41], leading to developmental delays, histological damage [43,44] and metamorphic failure [41]. For example, pyriproxyfen induces ultrastructural damage to midgut cells and behavioral anomalies in Dipteran and Lepidopteran models, associated with impaired cocooning and eclosion [20,42,43,44,45]. Similarly, OTFP, a JHE inhibitor, has been documented in *Dysdercus cingulatus* to induce the classic sequelae of developmental delays and malformed nymphs. In this context, our observation that OTFP treatment extends the nymphal period while simultaneously increasing pupal weight represents a counterintuitive departure from the established “negative paradigm,” highlighting the compensatory growth revealed in this study and its biological significance [42,46,47].

Our study demonstrates that the transcriptional response of *P. xylostella* to OTFP is a dynamic and multi-stage process, offering significant insights into the mechanisms of chemical endocrine disruptors. In the initial phase (24 h), a non-monotonic dose–response was observed, in which the low-concentration treatment induced more total DEGs than the high-concentration treatment, a recognized characteristic of endocrine disruptor studies [48]. Furthermore, the nature of this response differed markedly between concentrations. The high-concentration treatment triggered a clear adaptive stress suppression strategy [49], with down-regulated genes significantly outnumbering up-regulated ones. In contrast, the low-concentration treatment elicited a more balanced response, with nearly equal numbers of genes being up- and down-regulated (Figure 1A,B,E). This potent suppression at high concentration may initially be caused by broad inhibition of core molecular machinery due to cytotoxicity. However, a significant shift in response pattern occurred by 48 h, particularly under high-concentration exposure, where the transcriptional response transitioned from this suppression-dominated state to a large-scale, upregulation-dominated profile (Figure 1C–E). This shift suggests that the organism’s adaptive capacity may have reached a critical threshold [50].

The pronounced gene upregulation observed at 48 h is associated with the activation of key genes involved in detoxification and stress response, including numerous genes from the Cytochrome P450 (P450), Glutathione S-transferase (GST), and ABC transporter families (Figure 5), contributing to the metabolism and efflux of the xenobiotic compound [31]. The fundamental driver of these transcriptional changes likely stems from the disruption of juvenile hormone (JH) homeostasis caused by the persistent inhibition of JHE by OTFP. Therefore, the transcriptional reprogramming at 48 h serves as a molecular indicator of an overloaded detoxification system and reflects the physiological consequences of endocrine disruption. Finally, the minimal overlap in responsive genes between the 24 h and 48 h time points provides molecular evidence for a “two-stage response model,” indicating that the molecular mechanisms for coping with OTFP stress are highly time-specific. This finding lays the groundwork for our subsequent, more in-depth analysis of the key biological processes that predominate at each distinct stage. The foundation for this multi-stage adaptive response lies in the disruption of the canonical hormonal interplay that governs insect metamorphosis, which in turn triggers a cascade of compensatory and defensive mechanisms.

Insect metamorphosis is governed by the dynamic interplay between juvenile hormone (JH) and 20-hydroxyecdysone (20E), where the relative hormonal titers precisely orchestrate the timing and fidelity of developmental transitions [12,51]. In this process, JH maintains the larval state by inducing its primary transducer, *Krüppel-homolog 1* (*Kr-h1*), via the Met receptor, thereby preventing a precocious shift to pupation. Precise regulation of the JH titer is therefore critical, relying primarily on its rapid clearance at specific developmental stages by *juvenile hormone esterase* (JHE), a pivotal member of the insect carboxylesterase (CCE) superfamily [52]. As a canonical and potent JHE-specific inhibitor, OTFP directly disrupts JH metabolic homeostasis by targeting this crucial regulatory node [53].

Consistent with previous reports [26], OTFP treatment resulted in minimal acute lethality in *P. xylostella*, a resistance phenotype substantiated by our transcriptomic data. We observed a robust upregulation of multiple *cytochrome P450* (CYP) and *glutathione S-transferase* (GST) genes (Figure 5), with significant enrichment of xenobiotic and drug metabolism pathways. While this indicates the activation of a canonical Phase I and II detoxification network in response to chemical stress [31], we propose that this metabolic response is subordinate to a more sophisticated regulatory architecture. This regulatory architecture is built upon conserved transcriptional control systems, notably the CncC and nuclear receptor pathways, which function as master regulators of the xenobiotic stress response [54]. Recent evidence suggests that insects possess integrated circuits that couple endocrine homeostasis with stress adaptation. For example, Guo et al., discovered a regulatory loop in the *P. xylostella* midgut that modulates 20E signaling to coordinate fitness costs while conferring high-level resistance to Bt toxins [55]. This model, where hormonal signaling is intrinsically linked to xenobiotic defense, provides a compelling framework for our findings. Given that the primary impact of OTFP is the severe disruption of the endocrine system, we posit that the high survival rate of *P. xylostella* is not merely a result of direct detoxification. Instead, it is orchestrated by an analogous integrative mechanism that transduces the signal of endocrine imbalance into a coordinated upregulation of the detoxification machinery. This strategy ensures survival under severe hormonal stress, albeit at the cost of compromising the organism’s development.

A central, seemingly paradoxical finding of this study is that while OTFP inhibits JHE at the protein level, it concurrently induces transcriptional up-regulation of multiple *JHE/JHEH* genes (Figure 6), consistent with compensatory feedback to perturbed JH homeostasis. Although we did not directly quantify hemolymph JH titers, prior manipulative studies demonstrate that altering JHE activity changes in vivo JH availability/titer. In *Plutella xylostella*, CRISPR/Cas9 knockout of PxJHE elevates hemolymph JH titers across developmental stages and delays development [56]. In the same species, repression of *JHE* expression via METTL3/14-dependent m6A pathways likewise increases JH titer and modulates life-history traits [57]. Conversely, transgenic overexpression of *JHE* in *Bombyx mori* triggers precocious metamorphosis, consistent with reduced JH availability, whereas depletion (knockdown) of *JHE* extends larval growth [23,58]. Classic endocrine experiments further show that selective pharmacological inhibition of JHE sustains elevated JH action and delays metamorphosis [59]. In stark contrast to the activated degradation arm, JHAMT—a rate-limiting enzyme in JH biosynthesis—was transcriptionally suppressed after OTFP exposure, in line with feedback control of the corpora allata and the central role of JHAMT in JH production [60,61]. Taken together, these data provide a parsimonious explanation for the developmental delay under OTFP: by inhibiting JHE, OTFP likely shifts JH homeostasis toward higher effective JH, thereby postponing the larval-pupal transition; the observed compensatory up-regulation of *JHE/JHEH* and the concurrent repression of *JHAMT* (Figure 6) are both expected under this feedback. As elaborated in the following paragraph, the prolonged larval window is accompanied by metabolic reprogramming that supplies energy and precursors for biomass accumulation (Figure 3).

Building on the JHE–JH feedback and the developmental delay outlined above, the KEGG patterns in Figure 3 indicate that OTFP exposure triggers a coordinated metabolic reprogramming during the prolonged larval period rather than a simple suppression of growth. Pathways associated with anabolic metabolism—including amino acid biosynthesis and the central carbon metabolic network—were significantly enriched, providing precursors and energetic/reducing equivalents required for sustained tissue growth [62,63]. In parallel, enrichment of ribosome biogenesis and aminoacyl-tRNA biosynthesis indicates an upregulated translational capacity, a hallmark of increased protein synthesis needed for biomass deposition [64,65,66]. These coordinated metabolic changes offer a mechanistic explanation for the increased pupal biomass observed in OTFP-treated larvae despite delayed development. Rather than representing a pathological consequence alone, developmental delay can act as a physiological trade-off, whereby larvae extend the feeding window and convert additional time into biomass accumulation, consistent with the established framework of compensatory growth in animal systems [67,68,69,70,71,72,73].

## 5. Conclusions

This study reveals that *Plutella xylostella* larvae exhibit a complex non-monotonic dose response when exposed to the JHE inhibitor OTFP. Ultimately, they employ an unexpected physiological trade-off strategy, converting developmental delay into biomass accumulation via transcriptional remodeling of a key growth pathway. We demonstrate that the toxicological impact of OTFP is not limited to its canonical role as a JHE inhibitor; rather, its core mechanism involves eliciting an integrated physiological stress response encompassing metabolic compensation, signaling antagonism, and detoxification. This multifaceted response severely imbalances the critical hormonal crosstalk between the juvenile hormone (JH) and 20-hydroxyecdysone (20E) signaling pathways. At the tested concentration, this endocrine disruption manifested as low mortality but led to a prolonged time to pupation, alongside increased pupal duration and weight [26]. These findings provide novel mechanistic insights into how insect growth regulators (IGRs) compromise hormonal signaling integrity to ultimately impede insect development [74].

## Data Availability

The original contributions presented in this study are included in the article. Further inquiries can be directed to the corresponding authors.

## Supplementary Materials

The following supporting information can be downloaded at: https://www.mdpi.com/article/10.3390/insects16111152/s1, Table S1. Statistics of RNA-Seq data output and quality for each sample.rimer sequences of target genes used for qRT-PCR validation; Table S2. Primer sequences of target genes used for qRT-PCR validation.

## Abbreviations

The following abbreviations are used in this manuscript:

OTFP	3-octylthio-1,1,1-trifluoro-2-propanone
JH	Juvenile Hormone
20E	20-Hydroxyecdysone
JHE	Juvenile Hormone esterase
*P. xylostella*	*Plutella x**yl**ostella*
JHEH	Juvenile Hormone epoxide hydrolase
JHAMT	Juvenile Hormone acid O-methyltransferase
*Kr-h1*	*Krueppel homolog 1*
*EcR*	*Ecdysone receptor*
*BrC-Z4*	*Broad-complex isoform Z4*
*BrC-Z2*	*Broad complex isoform Z2*
*74EF*	*Ecdysone-induced protein 74EF*
*HR3*	*Hormone receptor HR3*
*CYP307A1*	*Cytochrome P450 307a1*
JHBP	Juvenile Hormone-binding protein
Met	Methoprene-tolerant
