# Supplementary material for: Transcriptomic Analysis of the Diamondback Moth Under Exposure to the Juvenile Hormone Esterase (JHE) Inhibitor 3-Octylthio-1,1,1-trifluoro-2-propanone (OTFP)
Source: Insects. 2025 Nov 11;16(11):1152. doi: 10.3390/insects16111152 (PMC12653607; doi:10.3390/insects16111152)
Supplement: Supplementary file 1 [file insects-16-01152-s001.zip › insects-3913825-supplementary.pdf]

Table S1. Statistics of RNA-Seq data output and quality for each sample.rimer sequences of target genes used for qRT-PCR validation.

Sample No.	Clean reads（bp）	GC Content(%)	Q30(%)	Mapped reads(%)
4CK1_1	53,289,416	52	95	72
4CK1_2	47,513,286	52	94	74
4CK1_3	49,479,180	52	94	74
4CK2_1	52,095,970	51	94	72
4CK2_2	48,406,106	49	95	70
4CK2_3	48,080,336	49	94	70
4TA1_1	59,112,636	52	94	73
4TA1_2	46,837,726	52	95	74
4TA1_3	47,994,686	51	95	74
4TA2_1	50,819,776	52	94	72
4TA2_2	49,919,810	51	94	73
4TA2_3	51,216,976	52	94	71
4TB1_1	49,493,060	51	95	72
4TB1_2	43,811,906	52	94	73
4TB1_3	46,655,074	52	95	77
4TB2_1	44,906,288	52	94	71
4TB2_2	54,095,256	51	94	72
4TB2_3	49,949,084	50	94	73

Notes: (1) Samples: The identifier for each analyzed sample. CK1_1~3 represent the re peated samples of the control group after treatment of 24 hours; CK2_1~3 represent the re peated samples of the control group after treatment of 48 hours; TA1_1~3 & TB1_1~3 re present the repeated samples of TA (treated with 1325 mg/L of OTFP) and TB (treated with 2 650 mg/L of OTFP) after treatment of 24 hours; TA2_1~3 & TB2_1~3 represent the repeated s amples of TA (treated with 1325 mg/L of OTFP) and TB (treated with 2650 mg/L of OTFP) after t reatment of 48 hours. (2) Clean reads: The total number of paired-end reads in the clean data. (3) Clean bases: The total number of bases in the clean data. (4) GC content: The percentage of Guanine (G) and Cytosine (C) bases among the total bases in the clean data. (5) Q30 (%): The percentage of bases with a Phred quality score of 30 or higher.

Table S2. Primer sequences of target genes used for qRT-PCR validation.

Genes No.	Description	Primer sequence
LOC105387899	Juvenile hormone esterase 1	F: CCCGCCTACCTCTACCAGTTC<br>R: ATTTTGCTGTCCGTGCTGTCC
LOC105387913	Juvenile hormone esterase 2	F: GTGTCCGTGGCTTCTTCAACT<br>R: AGGTGGGCAGTATGTTCTGGT
LOC105386617	juvenile hormone epoxide hydrolase 1	F: GCGACCTCTGACAAAACCTCT<br>R: CATCCTTCGGCTTGCTCCTAA
LOC105389233	juvenile hormone epoxide hydrolase 2	F: ACGTGATGGAGGAGTTCGGG<br>R: TCGGTACTGGGGTCTTGATGT
LOC105380497	Methoprene-tolerant	F: CGTTCGCTTTCGGGCATC<br>R: CTTGTAGGTGACGGTGAGGAG
LOC105392212	Krueppel homolog 1	F: CAAACTGCACCGCACGCAAC<br>R: CCACAGGGCACGTAGGAGAAG
LOC105394159	ecdysone receptor	F: GTGATGATGCTGCGAGTGGT<br>R: GCTCTGCTGGYTCAGGATGT
LOC105384337	broad-complex isoform Z4	F: TAGCCCGATCTCCACCGAC<br>R: TGTTGAGCGAGTTGAGCGTG
LOC105384337	broad complex isoform Z2	F: AGGCATATAGCGGACAAGCAC<br>R: GGCAGAGCTCGCACACATACA
LOC105393067	ecdysone-induced protein 74EF	F: AGCGACATCTCTTGGGGAACT<br>R: TTCAATCGGAGGCACAGACGA
LOC105394379	hormone receptor HR3	F: CCAGAACATCATCGAGTTTGCC<br>R: ACCACATCTCCATACAGAACCT
LOC105380847	cytochrome P450 307a1	F: CCATACACTGAAGCCACCATCC<br>R: TCGCCATCAGACTCCATTCCA
LOC105392916	juvenile hormone-binding protein	F: AAGGCGCTAGTGACAGTGAAG<br>R: CGGATGTTGTGGACGAAGGT
LOC105390789	elongation factor 1-beta	F:GCCTCCCTACAGCGAATC<br>R:CCTTGAACCAGGGCATCT

This table lists the detailed information of primers for 13 differentially expressed genes (DEGs) selected for qRT-PCR validation and elongation factor 1-beta is served as housekeeping gene. The table includes the Gene ID, Gene Name, and the forward and reverse primer sequences used for amplification
